# Investigating the relationship between psoriatic arthritis, multiple long-term conditions, treatment burden and adverse clinical outcomes: A systematic review protocol

**DOI:** 10.1177/26335565261459513

**Published:** 2026-07-03

**Authors:** George A. Opoku-Pare, Bhautesh D. Jani, Stefan Siebert, Frances S. Mair, Ilana Booth, Jordan Canning, Barbara I. Nicholl

**Affiliations:** 1 General Practice and Primary Care, School of Health and Wellbeing, 3526 University of Glasgow, College of Medical Veterinary and Life Sciences, UK; 2 Immunology and Inflammation, School of Infection and Immunity, 3526 University of Glasgow, College of Medical Veterinary and Life Sciences, UK; 3 Policy Modelling for Health Consortium, Public Health, School of Health and Wellbeing, 3526 University of Glasgow, College of Medical Veterinary and Life Sciences, UK

**Keywords:** psoriatic arthritis, multimorbidity, multiple long-term conditions, treatment burden, health-related outcomes

## Abstract

**Background:**

Psoriatic arthritis (PsA) is a chronic inflammatory musculoskeletal disease associated with psoriasis, affecting an estimated 0.13% of adults worldwide. People living with PsA often experience multiple long-term conditions (MLTCs) or multimorbidity (the presence of two or more long-term health conditions), such as hypertension, diabetes, obesity, and metabolic syndrome. Multimorbidity and treatment burden which is the workload of healthcare experienced by individuals and its impact on wellbeing may exacerbate poor health outcomes and complicate disease management in PsA. However, the influence of MLTCs and/or treatment burden on adverse health-related outcomes in this population remains poorly understood.

**Objective:**

This systematic review protocol will outline the methods to evaluate the current evidence regarding the impact, if any, of MLTCs and/or treatment burden on mortality and other adverse health-related outcomes in individuals with PsA.

**Design:**

Systematic review of the literature. The following databases will be searched: MEDLINE, EMBASE, CINAHL, PsycINFO, and Scopus. Longitudinal quantitative studies will be eligible for inclusion. Study selection will follow predefined eligibility criteria, and methodological quality and risk of bias will be assessed using the Cochrane Quality in Prognostic Studies tool. A narrative synthesis will be undertaken, and meta-analysis will be considered where appropriate. This protocol follows the Preferred Reporting Items for Systematic Reviews and Meta-Analyses Protocols (PRISMA-P) 2015 guidelines.

**Conclusions:**

Understanding the impact of MLTCs and/or treatment burden on health-related outcomes in PsA is essential for improving future clinical management. This review will help identify existing knowledge gaps and advance precision medicine strategies to improve health-related outcomes for individuals living with PsA.

## Introduction

### Rationale

Psoriatic arthritis (PsA) is an inflammatory musculoskeletal condition which is often associated with psoriasis, a predominantly dermatological condition.^1^ PsA prevalence is estimated at 0.13% across the globe^2,3^ and affects 20–30% of those with psoriasis.^4^ The persistent immune-mediated inflammation causes pain, swelling, tenderness, and gradual destruction to the synovial joints, that significantly impairs daily function and decreases quality of life.^5^ The onset of PsA is usually between the ages of 40 and 50 years^6^ and affects women and men approximately equally.^6,7^ Substantial increase in economic burden, increased morbidity and mortality, reduced productivity, exacerbated comorbidities and frequent hospitalizations are consequences of PsA.^8^ Improved understanding of the pathophysiology of PsA has led to the development and use of effective biologics and small-molecular drugs targeting specific cytokines and signalling pathways for patients with PsA.^5^ These drugs slow the progression of the disease and enhance quality of life.^5^ However, the presence of coexisting long-term conditions (LTCs) has the potential to worsen PsA symptoms and cause additional complications for individuals living with PsA.^9^

Comorbidity is highly prevalent among individuals with PsA, with studies reporting that over 90% of people with PsA have at least one additional LTC.^10^ The most common comorbid LTCs observed in PsA patients are hypertension (34.2%), metabolic syndrome (28.8%), obesity (27.4%), hyperlipidaemia (24.2%) and diabetes mellitus (12.9%).^11^ Labitigan et al.^12^ conducted a cross-sectional study including 3,132 PsA patients and, after a 10-year follow-up, reported that patients with PsA had a higher prevalence of cardiometabolic diseases compared to those with rheumatoid arthritis (RA); prevalence of obesity was 48% in PsA and 37%, respectively, diabetes 12% and 8% respectively.

According to estimates, 40% of people with PsA will have three or more LTCs.^13^ Multimorbidity (the coexistence of two or more LTCs in an individual),^14^ is associated with higher mortality risk, lower quality of life, and increased healthcare utilisation in people living with PsA.^15,16^ Treatment burden, the workload of healthcare experienced by those living with LTCs and the impact that this has on wellbeing, is increased for people living with multimorbidity and ability to manage this workload varies with an individual’s capacity and networks.^17^ For individuals with PsA, this treatment burden workload is compounded by frequent healthcare utilization, and the complex demands of managing coexisting multiple long-term conditions (MLTCs).^18,19^

However, the implications of having PsA alongside other MLTCs is poorly understood. This systematic review will examine how MLTCs and/or treatment burden influence health-related outcomes in people with PsA. Synthesising the evidence in this area will help underpin future work aimed at improving the management and quality of life for people living with PsA and MLTCs.

### Objectives

This protocol outlines the methods for a systematic review that aims to evaluate the current evidence of the impact, if any, of coexisting MLTCs or treatment burden on the risk of mortality and other adverse health related outcomes including hospitalizations, functional status and health-related quality of life in individuals with PsA.

## Methods

The systematic review will be conducted in accordance with the Preferred Reporting Items for Systematic Reviews and Meta-Analyses Protocols (PRISMA-P) 2015 reporting guidelines^20^ (Online Supplementary File S1) has been registered in PROSPERO (CRD420251162364).

### Eligibility criteria

PICO framework^21^ has been used to structure eligibility criteria adapted to PECOS,^22^ Population, Exposure, Comparator, Outcomes and additionally study design. These criteria are summarised in Table 1.

**Table 1.** Summary of inclusion and exclusion criteria for the study eligibility.

Category	Inclusion criteria	Exclusion criteria
Population	Adults (≥18 years) with PsA, with or without psoriasis)	Children/adolescents (<18 years old), studies that cannot differentiate between psoriasis and PsA, studies focused on the effect of drug treatment for PsA only, and animal studies.
Exposure or intervention	Presence of multimorbidity/Multiple long-term conditions: defined as the “co-existence of ≥2 long-term physical or mental health conditions in an individual” alongside PsA.	Any studies with PsA ≤1 LTC
	And/OR	
	Treatment burden: The workload or demand placed on the patient as a consequence of managing their health.	
Comparison or control	No comparison or control group will be required for study inclusion. But where a comparison group of non-PsA individuals is included, it will be reported.	Not Applicable
Outcomes	Mortality, health-related outcomes, quality of life, mental health and major adverse cardiovascular events (MACE) and patient reported outcomes.	​
Publication type	Full text published article	Conference, abstracts; dissertations/theses; editorials/commentaries/letters
Language	English language	Non – English language
Study design	Longitudinal cohort studies (prospective and retrospective)	Cross-sectional studies; intervention studies; qualitative studies, case reports/series; and review articles (including systematic reviews)

#### Population

Studies must include adults (≥18 years) diagnosed with PsA, with or without psoriasis, and presenting with two or more additional physical or mental health long-term conditions (LTCs). Exclusion criteria comprise studies involving children or adolescents (<18 years), animal studies, studies that do not distinguish between psoriasis and PsA, studies including participants with PsA and only one LTC, and studies examining solely the effects of drug treatment for PsA.

#### Exposure

The exposures of interest are multimorbidity/MLTCs, defined as the co-existence of two or more LTCs, and/or treatment burden, defined as the workload or demands placed on patients in managing their health, among adults with PsA. We will include studies that assess the relationship between PsA, MLTCs and/or treatment burden, and our outcomes of interest using any numerical measure of MLTCs, provided the types of comorbid conditions are specified. Studies focusing solely on one comorbid condition in individuals with PsA will be excluded, as this review is specifically concerned with the relationship between PsA, MLTCs, and/or treatment burden.

#### Comparators

The inclusion of a comparator or control group is not mandatory for study eligibility, as it is not essential to address the review question. However, where one has been reported, we will include it in our findings. The most applicable comparator group would be adults with two or more long-term physical or mental health conditions without PsA. Alternatively, the comparator may include individuals with RA and two or more additional LTCs, assessed for the same general outcomes (e.g., mortality, health-related outcomes).

#### Outcomes

The primary outcomes will be all-cause mortality, health-related outcomes, quality of life, mental health, and major adverse cardiovascular events (MACE) in individuals with PsA. Patient-reported outcome measures of physical, emotional, and social wellbeing, assessed through validated health assessment questionnaires and other patient-reported outcome tools, will be considered as secondary outcomes. Studies reporting data on any of these outcomes will be eligible for inclusion.

#### Study design

Our review will include empirical studies that employ quantitative methodologies. We will specifically focus on longitudinal cohort studies, encompassing both retrospective and prospective designs. Studies utilizing cross-sectional studies, interventional designs, qualitative studies, case reports or series, and review articles (including systematic reviews) will be excluded.

#### Publication type

Eligible studies will be limited to full text, published articles. Conference abstracts, dissertations or theses, and editorial materials, including commentaries and letters, will be excluded.

#### Language

Studies must be in English language.

### Data source and search strategy

The following electronic medical databases will be utilised: MEDLINE (accessed via Ovid), EMBASE (accessed via Ovid), Cumulative Index of Nursing and Allied Health (CINAHL; accessed via EBSCOhost), PsycINFO (accessed via EBSCOhost) and Scopus (accessed via Elsevier). The search strategy will combine the following concepts: (1) psoriatic arthritis, AND (2) multimorbidity/multiple long-term conditions, OR (3) treatment burden AND (4) mortality, OR (5) health-related outcomes (view ‘Outcomes’ section), using subject index terms and keywords(Table 2). Advanced search techniques, including multi-field searches, operators, truncation/wildcards, and limits, will be used in combination with appropriate Boolean terms to develop the search strategy. Each database will be searched individually with the search strategy adapted to reflect the differing subject index terms and keywords used by each database. The search strategy will be evaluated by the University of Glasgow’s College Librarian for Medical, Veterinary and Life Sciences and by review panel members with expertise in PsA and multimorbidity (Online Supplementary File S2).

**Table 2.** Summary of subject index terms and keywords used in search strategy for electronic medical database.

Key concepts	Psoriatic arthritis	Multimorbidity	Treatment burden	Mortality	Health-related outcomes
Search index terms and keywords	Psoriatic Arthritis/exp Arthritis/AND Psoriasis/Enthesopathy/psoriat$.ti,ab,kw. enthesitis.ti,ab,kw. enthesopath$.ti,ab,kw. dactylitis.ti,ab,kw. ((psorias$ or nail$) adj5 (arthrit$ or arthropath$ or monoarthr$ or oligoarthr$ or polyarthr$ or rheumat$)).ti,ab,kw. ((psorias$ or nail$) adj5 (axial or peripheral or spondyl?arthr$ or arthropath$ or spondylitis or sacroili?ti$)).ti,ab,kw. (psorias$ or nail$).ti,ab,kw. AND (spondyloarthropathy/OR Spondylarthritis/OR spondylitis ankylosing/OR sacroiliitis/) axPsA.ti,ab,kw. pPsA.ti,ab,kw. “psoriatic arthritis”.ti,ab. “arthritis psoriatica”.ti,ab. “psoriatic arthropathy”.ti,ab. “psoriasis arthropathica”.ti,ab. “arthritic psoriasis”.ti,ab. (psoriatic adj1 arthritis).ti,ab	Multimorbid* OR multi-morbid* OR comorbidit* OR co-morbidit* OR polymorbidit* OR poly-morbidit* OR multi-condition* OR multicondition* OR ((multiple OR coexist* OR co-exist* OR concurrent OR co-occurr* OR comorbid* OR co-morbid*) ADJ1 (“long term” OR long-term OR chronic OR diseas* OR illness* OR morbid* OR condition*))).tw.	(‘treatment burden'/exp OR ‘treatment burden':ti,ab OR ‘burden of treatment':ti,ab OR ‘medication burden':ti,ab OR ‘therapeutic burden':ti,ab OR ‘patient burden':ti,ab OR ‘caregiver burden':ti,ab OR ‘treatment-related burden':ti,ab OR ‘care burden':ti,ab OR ‘treatment adherence burden':ti,ab OR ‘healthcare burden':ti,ab OR ‘disease management burden':ti,ab OR ‘compliance burden':ti,ab)	mortalit* OR death* OR “cause of death” OR “all-cause mortality” OR “disease-specific mortality” OR fatalit* OR surviv* OR “time to death”	(‘health outcome'/exp OR ‘health outcome':ti,ab OR ‘quality of life':ti,ab OR ‘health status':ti,ab OR ‘patient outcome':ti,ab OR ‘treatment outcome':ti,ab OR ‘clinical outcome':ti,ab OR ‘disease outcome':ti,ab OR ‘functional outcome':ti,ab OR ‘health care outcome':ti,ab OR ‘well-being':ti,ab) OR ‘patient reported outcome measures':ti,ab OR ‘patient reported outcome':ti,ab OR ‘patient-reported outcome':ti,ab OR ‘patient reported outcomes':ti,ab OR ‘function':ti,ab OR ‘pain'/exp OR ‘pain':ti,ab OR ‘chronic pain'/exp OR ‘chronic pain':ti,ab OR ‘health assessment questionnaire'/exp OR ‘health assessment questionnaire':ti,ab

### Data screening and extraction

#### Data management

The search results from the five main databases will be exported to EndNote (version 21.5; Clarivate) for deduplication. The deduplicated records will then be imported into the systematic review management platform, DistillerSR (Evidence Partners, Ontario, Canada), to support the study selection process and a further duplication check will be run.

#### Screening process

Studies will undergo a two-stage screening process in DistillerSR. Titles and abstracts will first be screened independently by the primary researcher (GAOP) and another member of the review team (BN, IB or JC) against predefined eligibility criteria. All conflicts in title and abstract screening will be resolved by BN. Studies that meet the criteria will proceed to full-text screening. Full-text reports will be obtained and reviewed independently by the primary researcher (GAOP) and other reviewers (IB or JC), with supplementary material consulted as needed to support the decision-making process. Disagreements at this stage will be resolved through arbitration by a third reviewer (BN). Citation searching of included full texts will also be conducted to identify additional relevant studies.

#### Data extraction process

Data extraction will be conducted using a piloted form based on the study PECOS.^22^ Two reviewers will extract data independently, with discrepancies resolved by discussion or consultation with a third reviewer. The form will capture population, exposure, comparator, outcomes, study design, and key study characteristics such as setting, period, source of data, aims and objectives and results.

### Specifics of data extraction

#### Study characteristics

Information on study design, setting, study period, aims and objectives will be extracted.

#### Population

Key characteristics of the study populations, including sex, ethnicity, sample size, age, sociodemographic factors, occupation, PsA duration, treatment status and disease activity will be collected where available. Recruitment methods, sampling techniques, and inclusion/exclusion criteria applied in each study will be recorded. Also, the diagnostic criteria or case definition used for PsA will be documented.

#### Exposure

Definitions and measures of multimorbidity/MLTCs will be extracted, including the number, type, and classification of conditions reported in each study. Measures of treatment burden will also be collected (e.g. Patient Experience with Treatment and Self-Management (PETS), where available.

#### Comparator

Details of comparator or control groups will be extracted, including how they are defined.

#### Outcome

Information on outcome definitions and measurement will be collected, including all-cause mortality, health-related outcomes (e.g., quality of life, functional status, pain, patient-reported outcomes), and major adverse cardiovascular events (MACE). Data on follow-up duration, outcome ascertainment, and the statistical approaches used to evaluate associations between PsA, multimorbidity, treatment burden, and these outcomes will also be extracted. The primary outcome is all-cause mortality, which may be reported as odds ratios, incidence rates, hazard ratios, or survival percentages. Health-related outcomes will include both clinician and patient-reported measures such as Health Assessment Questionnaire (HAQ) and patient/physician global assessments.

#### Statistical methods

Information on the statistical methods employed in each study will be extracted, including the analytic approaches used, reported effect sizes, and measures of association. Details of model adjustments, including the confounders accounted for, will also be documented. If the literature allows, meta-analysis will be performed using R software (RStudio), employing the meta and metafor packages for statistical synthesis, heterogeneity assessment, subgroup analyses, and sensitivity analyses.

#### Risk of bias (quality) assessment

The risk of bias of included studies will be independently evaluated by two reviewers using the Quality In Prognosis Studies (QUIPS) tool,^23,24^developed by the Cochrane Prognosis Methods Group. Assessments will be conducted across six domains: study participation, study attrition, prognostic factor measurement, outcome measurement, study confounding, and statistical analysis and reporting. Each domain will be assigned a risk-of-bias rating of low, moderate, or high. The adequacy of reporting within each domain will be rated as yes, partial, no, or unsure. Text comments will be documented as needed to support consensus discussions. Any discrepancies between reviewers will be resolved through discussion, with consultation of a third reviewer if consensus cannot be reached.

#### Data synthesis

Studies will be grouped by outcomes, and the narrative synthesis will summarise study characteristics, setting and sample size, population, exposures and comparators, outcomes, study design,risk of bias, and any inconsistencies. Where feasible, a meta-analysis will be conducted following an assessment of heterogeneity and publication bias. Subgroup analyses may be undertaken if sufficient data are available, based on study population characteristics such as age, gender, socioeconomic status, or MLTC profile, including the count, type, and classification of comorbid conditions.

### Ethical approval and dissemination

As this systematic review will synthesise data from previously published studies and will not involve the collection of individual patient-level data, formal ethics approval is not required. The findings of the review will be disseminated through multiple channels, including presentations at relevant scientific conferences, publication in peer-reviewed journals, and communication via professional social media platforms to reach a broader audience. Additionally, the completion of this review contributes to the objectives of the primary researcher’s PhD project, providing a foundation for further research and knowledge translation in the field of precision medicine.

### Patient and public involvement

Although this systematic review will not directly involve patients or the public in its conduct, its focus was shaped by substantial treatment burden and complex healthcare utilizations experienced by individuals living with PsA and MLTCs. The review acknowledges that managing concurrent conditions, frequent healthcare appointments and admissions can significantly impact wellbeing and quality of life. Our findings will be shared with patient representatives from PsA support groups and public involvement network for feedback. This process will help align the results with the lived experiences and inform practical recommendations for clinical care and policy.

## Discussion

This protocol outlines a systematic review that will examine existing literature to determine the current evidence regarding the impact, if any, of MLTCs and/or treatment burden on adverse health-related outcomes and mortality in individuals with PsA. We hypothesize that the presence of MLTCs or treatment burden is associated with higher risk of mortality, adverse health-related outcomes and reduced quality of life among people living with PsA. The purpose of this systematic review is to explore how coexisting MLTCs and/or treatment burden influence the risk of mortality and other adverse health-related outcomes including hospitalisations, functional status and quality of life in individuals with PsA.

### What this adds to the literature

To our knowledge, this systematic review represents the first effort to comprehensively examine and synthesise the current evidence on the impact of MLTCs and/or treatment burden, for people living with PsA. By elucidating these relationships, the review aims to identify gaps in existing knowledge and advance understanding of how MLTCs and treatment burden influence health outcomes for people with PsA. Highlighting these areas for further investigation may help bridge the gap between current evidence and the development of future clinical guidelines or health service interventions that could more effectively support patients with PsA and coexisting LTCs. In doing so, this review has the potential to inform precision medicine approach to PsA management by stratifying patients at highest risk of adverse clinical and health-related outcomes.

## Strengths

This review has several methodological strengths. It will be conducted in accordance with the PRISMA-P 2015 guidelines, ensuring transparency, clarity, and reproducibility in reporting. A comprehensive search strategy will be formulated in consultation with a professional librarian to minimise the risk of omitting relevant studies. In addition, all study screening and data extraction will be undertaken independently by two reviewers, thereby reducing the potential for selection bias and oversight. By assessing both all-cause mortality, health-related outcomes, quality of life, mental health, and MACE this review will provide a holistic appraisal of MLTCs or treatment burden among individuals with PsA.

## Limitations

Nevertheless, some methodological limitations should be taken into consideration. Variability in the definitions and measurements of MLTCs, treatment burden and adverse health-related outcomes is anticipated across studies, which may introduce heterogeneity and constrain the feasibility of quantitative synthesis. Where meta-analysis is not appropriate, a narrative synthesis will be employed to accommodate the heterogeneity of the literature. Furthermore, the search will be limited to studies published in English, potentially excluding relevant evidence in other languages. However, existing evidence suggests that language restrictions are unlikely to substantially influence the conclusions of systematic reviews.^25^

## Conclusion

In summary, without a rigorous understanding of how the association of PsA with the presence of MLTCs and/or treatment burden influences the risk of mortality and adverse health related outcomes, the development of personalized treatment approaches and clinical guidelines for this complex PsA population remains challenging. By advancing comprehension of these relationships, this review has the potential to support risk stratification for individuals with PsA (Precision Medicine) who are at higher risk of adverse clinical and health-related outcomes.

## Supplemental material

Supplemental material - Investigating the relationship between psoriatic arthritis, multiple long-term conditions, treatment burden and adverse clinical outcomes: A systematic review protocols Supplemental material for Investigating the relationship between psoriatic arthritis, multiple long-term conditions, treatment burden and adverse clinical outcomes: A systematic review protocol by George A. Opoku-Pare, Bhautesh D. Jani, Stefan Siebert, Frances S. Mair, Ilana Booth, Jordan Canning, Barbara I. Nicholl in Journal of Multimorbidity and Comorbidity.

Supplemental material - Investigating the relationship between psoriatic arthritis, multiple long-term conditions, treatment burden and adverse clinical outcomes: A systematic review protocols Supplemental material for Investigating the relationship between psoriatic arthritis, multiple long-term conditions, treatment burden and adverse clinical outcomes: A systematic review protocol by George A. Opoku-Pare, Bhautesh D. Jani, Stefan Siebert, Frances S. Mair, Ilana Booth, Jordan Canning, Barbara I. Nicholl in Journal of Multimorbidity and Comorbidity.

## Data Availability

Template data collection forms, data extracted from included studies, data used for all analyses, and analytic code will be available in the published systematic review, as online supplementary material, or in GitHub.*
