# Supplementary material for: Investigating the relationship between psoriatic arthritis, multiple long-term conditions, treatment burden and adverse clinical outcomes: A systematic review protocol
Source: J Multimorb Comorb. 2026 Jul 3;16:26335565261459513. doi: 10.1177/26335565261459513 (PMC13332292; doi:10.1177/26335565261459513)
Supplement: Supplemental material - Investigating the relationship between psoriatic arthritis, multiple long-term conditions, treatment burden and adverse clinical outcomes: A systematic review protocols [file sj-pdf-1-cob-10.1177_26335565261459513.pdf]

Supplementary File S2: Full Search Strategy Draft for Multiple Databases

MEDLINE

Search No.	Subject index terms and keywords
1	Psoriatic Arthritis/
2	exp Arthritis/ AND Psoriasis/
3	Enthesopathy/
4	psoriat$.ti,ab,kw.
5	enthesitis.ti,ab,kw.
6	enthesopath$.ti,ab,kw.
7	dactylitis.ti,ab,kw.
8	((psorias$ or nail$) adj5 (arthrit$ or arthropath$ or monoarthr$ or oligoarthr$ or polyarthr$ or rheumat$)).ti,ab,kw.
9	((psorias$ or nail$) adj5 (axial or peripheral or spondyl?arthr$ or arthropath$ or spondylitis or sacroili?ti$)).ti,ab,kw.
10	(psorias$ or nail$).ti,ab,kw. AND (spondyloarthropathy/ OR Spondylarthritis/ OR spondylitis ankylosing/ OR sacroiliitis/)
11	axPsA.ti,ab,kw.
12	pPsA.ti,ab,kw.
13	"psoriatic arthritis".ti,ab.
14	"arthritis psoriatica".ti,ab.
15	"psoriatic arthropathy".ti,ab.
16	"psoriasis arthropathica".ti,ab.
17	"arthritic psoriasis".ti,ab.
18	(psoriatic adj1 arthritis).ti,ab.
19	1 or 2 or 3 or 4 or 5 or 6 or 7 or 8 or 9 or 10 or 11 or 12 or 13 or 14 or 15 or 16 or 17 or 18
20	Multimorbid* OR multi-morbid* OR comorbidit* OR co-morbidit* OR polymorbidit* OR poly-morbidit* OR multi-condition* OR multicondition* OR ((multiple OR coexist* OR co-exist* OR concurrent OR co-occurr* OR comorbid* OR co-morbid*) ADJ1 ("long term" OR long-term OR chronic OR diseas* OR illness* OR morbid* OR condition*)).tw.
21	20
22	('treatment burden'/exp OR 'treatment burden':ti,ab OR 'burden of treatment':ti,ab OR 'medication burden':ti,ab OR 'therapeutic burden':ti,ab OR 'patient burden':ti,ab OR 'caregiver burden':ti,ab OR 'treatment-related burden':ti,ab OR 'care burden':ti,ab OR 'treatment adherence burden':ti,ab OR 'healthcare burden':ti,ab OR 'disease management burden':ti,ab OR 'compliance burden':ti,ab)
23	mortalit* OR death* OR "cause of death" OR "all-cause mortality" OR "disease-specific mortality" OR fatalit* OR surviv* OR "time to death"
24	('health outcome'/exp OR 'health outcome':ti,ab OR 'quality of life':ti,ab OR 'health status':ti,ab OR 'patient outcome':ti,ab OR 'treatment outcome':ti,ab OR 'clinical outcome':ti,ab OR 'disease outcome':ti,ab OR 'functional outcome':ti,ab OR 'health care outcome':ti,ab OR 'well-being':ti,ab) OR 'patient reported outcome measures':ti,ab OR 'patient reported outcome':ti,ab OR 'patient-reported outcome':ti,ab OR 'patient reported outcomes':ti,ab OR 'function':ti,ab OR 'pain'/exp OR 'pain':ti,ab OR 'chronic pain'/exp OR 'chronic pain':ti,ab OR 'health assessment questionnaire'/exp OR 'health assessment questionnaire':ti,ab

25	19 and 21 and 22 and 23 and 24
26	limit 25 to English language

EMBASE

Search No.	Subject index terms and keywords
1	Psoriatic Arthritis/
2	exp Arthritis/ AND Psoriasis/
3	Enthesopathy/
4	psoriat$.ti,ab.
5	enthesitis.ti,ab.
6	enthesopath$.ti,ab.
7	dactylitis.ti,ab.
8	((psorias$ or nail$) NEAR/5 (arthrit$ or arthropath$ or monoarthr$ or oligoarthr$ or polyarthr$ or rheumat$)).ti,ab.
9	((psorias$ or nail$) NEAR/5 (axial or peripheral or spondyl?arthr$ or arthropath$ or spondylitis or sacroili?ti$)).ti,ab.
10	(psorias$ or nail$).ti,ab. AND (spondyloarthropathy/ OR Spondylarthritis/ OR spondylitis ankylosing/ OR sacroiliitis/)
11	axPsA.ti,ab.
12	pPsA.ti,ab.
13	"psoriatic arthritis".ti,ab.
14	"arthritis psoriatica".ti,ab.
15	"psoriatic arthropathy".ti,ab.
16	"psoriasis arthropathica".ti,ab.
17	"arthritic psoriasis".ti,ab.
18	(psoriatic adj1 arthritis).ti,ab.
19	1 or 2 or 3 or 4 or 5 or 6 or 7 or 8 or 9 or 10 or 11 or 12 or 13 or 14 or 15 or 16 or 17 or 18
20	Multimorbid* OR multi-morbid* OR comorbidit* OR co-morbidit* OR polymorbidit* OR poly-morbidit* OR multi-condition* OR multicondition* OR ((multiple OR coexist* OR co-exist* OR concurrent OR co-occurr* OR comorbid* OR co-morbid*) NEAR/1 ("long term" OR long-term OR chronic OR diseas* OR illness* OR morbid* OR condition*)).tw.
21	20
22	('treatment burden'/exp OR 'treatment burden':ti,ab OR 'burden of treatment':ti,ab OR 'medication burden':ti,ab OR 'therapeutic burden':ti,ab OR 'patient burden':ti,ab OR 'caregiver burden':ti,ab OR 'treatment-related burden':ti,ab OR 'care burden':ti,ab OR 'treatment adherence burden':ti,ab OR 'healthcare burden':ti,ab OR 'disease management burden':ti,ab OR 'compliance burden':ti,ab)
23	mortalit* OR death* OR "cause of death" OR "all-cause mortality" OR "disease-specific mortality" OR fatalit* OR surviv* OR "time to death"
24	('health outcome'/exp OR 'health outcome':ti,ab OR 'quality of life':ti,ab OR 'health status':ti,ab OR 'patient outcome':ti,ab OR 'treatment outcome':ti,ab OR 'clinical outcome':ti,ab OR 'disease outcome':ti,ab OR 'functional outcome':ti,ab OR 'health care outcome':ti,ab OR 'well-being':ti,ab) OR 'patient reported outcome measures':ti,ab OR 'patient reported outcome':ti,ab OR 'patient-reported outcome':ti,ab OR 'patient reported outcomes':ti,ab OR 'function':ti,ab OR 'pain'/exp OR 'pain':ti,ab OR 'chronic pain'/exp OR 'chronic pain':ti,ab OR 'health assessment questionnaire'/exp OR 'health assessment questionnaire':ti,ab
25	19 and 21 and 22 and 23 and 24
26	limit 25 to English language

PsycINFO

Search No.	Subject index terms and keywords
1	'Psoriatic Arthritis'
2	'Arthritis' AND 'Psoriasis'
3	Enthesopathy/
4	psoriat$.ti,ab.
5	enthesitis.ti,ab.
6	enthesopath$.ti,ab.
7	dactylitis.ti,ab.
8	((psorias$ or nail$) NEAR/5 (arthrit$ or arthropath$ or monoarthr$ or oligoarthr$ or polyarthr$ or rheumat$)).ti,ab.
9	((psorias$ or nail$) NEAR/5 (axial or peripheral or spondyl?arthr$ or arthropath$ or spondylitis or sacroili?ti$)).ti,ab.
10	(psorias$ or nail$).ti,ab. AND (spondyloarthropathy/ OR Spondylarthritis/ OR spondylitis ankylosing/ OR sacroiliitis/)
11	axPsA.ti,ab.
12	pPsA.ti,ab.
13	"psoriatic arthritis".ti,ab.
14	"arthritis psoriatica".ti,ab.
15	"psoriatic arthropathy".ti,ab.
16	"psoriasis arthropathica".ti,ab.
17	"arthritic psoriasis".ti,ab.
18	(psoriatic adj1 arthritis).ti,ab.
19	1 or 2 or 3 or 4 or 5 or 6 or 7 or 8 or 9 or 10 or 11 or 12 or 13 or 14 or 15 or 16 or 17 or 18
20	Multimorbid* OR multi-morbid* OR comorbidit* OR co-morbidit* OR polymorbidit* OR poly-morbidit* OR multi-condition* OR multicondition* OR ((multiple OR coexist* OR co-exist* OR concurrent OR co-occurr* OR comorbid* OR co-morbid*) NEAR/1 ("long term" OR long-term OR chronic OR diseas* OR illness* OR morbid* OR condition*)).tw.
21	20
22	('treatment burden' OR 'treatment burden':ti,ab OR 'burden of treatment':ti,ab OR 'medication burden':ti,ab OR 'therapeutic burden':ti,ab OR 'patient burden':ti,ab OR 'caregiver burden':ti,ab OR 'treatment-related burden':ti,ab OR 'care burden':ti,ab OR 'treatment adherence burden':ti,ab OR 'healthcare burden':ti,ab OR 'disease management burden':ti,ab OR 'compliance burden':ti,ab)
23	mortalit* OR death* OR "cause of death" OR "all-cause mortality" OR "disease-specific mortality" OR fatalit* OR surviv* OR "time to death"
24	('health outcome' OR 'health outcome':ti,ab OR 'quality of life':ti,ab OR 'health status':ti,ab OR 'patient outcome':ti,ab OR 'treatment outcome':ti,ab OR 'clinical outcome':ti,ab OR 'disease outcome':ti,ab OR 'functional outcome':ti,ab OR 'health care outcome':ti,ab OR 'well-being':ti,ab) OR 'patient reported outcome measures':ti,ab OR 'patient reported outcome':ti,ab OR 'patient-reported outcome':ti,ab OR 'patient reported outcomes':ti,ab OR 'function':ti,ab OR 'pain' OR 'pain':ti,ab OR 'chronic pain' OR 'chronic pain':ti,ab OR 'health assessment questionnaire' OR 'health assessment questionnaire':ti,ab
25	19 and 21 and 22 and 23 and 24
26	limit 25 to English language

CINAHL

Search No.	Subject index terms and keywords
1	MH 'Psoriatic Arthritis+'
2	MH 'Arthritis*' AND MH 'Psoriasis*'
3	Enthesopathy/
4	psoriat$.TI,AB.
5	enthesitis.TI,AB.
6	enthesopath$.TI,AB.
7	dactylitis.TI,AB.
8	((psorias$ or nail$) N5 (arthrit$ or arthropath$ or monoarthr$ or oligoarthr$ or polyarthr$ or rheumat$)).TI,AB.
9	((psorias$ or nail$) N5 (axial or peripheral or spondyl?arthr$ or arthropath$ or spondylitis or sacroili?ti$)).TI,AB.
10	(psorias$ or nail$).TI,AB. AND (spondyloarthropathy/ OR Spondylarthritis/ OR spondylitis ankylosing/ OR sacroiliitis/)
11	axPsA.TI,AB.
12	pPsA.TI,AB.
13	"psoriatic arthritis".ti,ab.
14	"arthritis psoriatica".ti,ab.
15	"psoriatic arthropathy".ti,ab.
16	"psoriasis arthropathica".ti,ab.
17	"arthritic psoriasis".ti,ab.
18	(psoriatic adj1 arthritis).ti,ab.
19	1 or 2 or 3 or 4 or 5 or 6 or 7 or 8 or 9 or 10 or 11 or 12 or 13 or 14 or 15 or 16 or 17 or 18
20	Multimorbid* OR multi-morbid* OR comorbidit* OR co-morbidit* OR polymorbidit* OR poly-morbidit* OR multi-condition* OR multicondition* OR ((multiple OR coexist* OR co-exist* OR concurrent OR co-occurr* OR comorbid* OR co-morbid*) N1 ("long term" OR long-term OR chronic OR diseas* OR illness* OR morbid* OR condition*)).tw.
21	20
22	('treatment burden'+ OR 'treatment burden':ti,ab OR 'burden of treatment':ti,ab OR 'medication burden':ti,ab OR 'therapeutic burden':ti,ab OR 'patient burden':ti,ab OR 'caregiver burden':ti,ab OR 'treatment-related burden':ti,ab OR 'care burden':ti,ab OR 'treatment adherence burden':ti,ab OR 'healthcare burden':ti,ab OR 'disease management burden':ti,ab OR 'compliance burden':ti,ab)
23	mortalit* OR death* OR "cause of death" OR "all-cause mortality" OR "disease-specific mortality" OR fatalit* OR surviv* OR "time to death"
24	('health outcome'+ OR 'health outcome':ti,ab OR 'quality of life':ti,ab OR 'health status':ti,ab OR 'patient outcome':ti,ab OR 'treatment outcome':ti,ab OR 'clinical outcome':ti,ab OR 'disease outcome':ti,ab OR 'functional outcome':ti,ab OR 'health care outcome':ti,ab OR 'well-being':ti,ab) OR 'patient reported outcome measures':ti,ab OR 'patient reported outcome':ti,ab OR 'patient-reported outcome':ti,ab OR 'patient reported outcomes':ti,ab OR 'function':ti,ab OR 'pain'+ OR 'pain':ti,ab OR 'chronic pain'+ OR 'chronic pain':ti,ab OR 'health assessment questionnaire'+ OR 'health assessment questionnaire':ti,ab
25	19 and 21 and 22 and 23 and 24
26	limit 25 to English language

Scopus

Search No.	Subject index terms and keywords
1	TITLE-ABS-KEY(Psoriatic Arthritis/)
2	TITLE-ABS-KEY(exp Arthritis/ AND Psoriasis/)
3	TITLE-ABS-KEY(Enthesopathy/)
4	TITLE-ABS-KEY(psoriat$)
5	TITLE-ABS-KEY(enthesitis)
6	TITLE-ABS-KEY(enthesopath$)
7	TITLE-ABS-KEY(dactylitis)
8	TITLE-ABS-KEY(((psorias$ or nail$) W/5 (arthrit$ or arthropath$ or monoarthr$ or oligoarthr$ or polyarthr$ or rheumat$)))
9	TITLE-ABS-KEY(((psorias$ or nail$) W/5 (axial or peripheral or spondyl?arthr$ or arthropath$ or spondylitis or sacroili?ti$)))
10	TITLE-ABS-KEY((psorias$ or nail$) AND (spondyloarthropathy/ OR Spondylarthritis/ OR spondylitis ankylosing/ OR sacroiliitis/))
11	TITLE-ABS-KEY(axPsA)
12	TITLE-ABS-KEY(pPsA)
13	TITLE-ABS-KEY("psoriatic arthritis".ti,ab.)
14	TITLE-ABS-KEY("arthritis psoriatica".ti,ab.)
15	TITLE-ABS-KEY("psoriatic arthropathy".ti,ab.)
16	TITLE-ABS-KEY("psoriasis arthropathica".ti,ab.)
17	TITLE-ABS-KEY("arthritic psoriasis".ti,ab.)
18	TITLE-ABS-KEY((psoriatic adj1 arthritis).ti,ab.)
19	1 or 2 or 3 or 4 or 5 or 6 or 7 or 8 or 9 or 10 or 11 or 12 or 13 or 14 or 15 or 16 or 17 or 18
20	TITLE-ABS-KEY(Multimorbid* OR multi-morbid* OR comorbidit* OR co-morbidit* OR polymorbidit* OR poly-morbidit* OR multi-condition* OR multicondition* OR ((multiple OR coexist* OR co-exist* OR concurrent OR co-occurr* OR comorbid* OR co-morbid*) W/1 ("long term" OR long-term OR chronic OR diseas* OR illness* OR morbid* OR condition*)).tw.)
21	20
22	TITLE-ABS-KEY(('treatment burden' OR 'treatment burden':ti,ab OR 'burden of treatment':ti,ab OR 'medication burden':ti,ab OR 'therapeutic burden':ti,ab OR 'patient burden':ti,ab OR 'caregiver burden':ti,ab OR 'treatment-related burden':ti,ab OR 'care burden':ti,ab OR 'treatment adherence burden':ti,ab OR 'healthcare burden':ti,ab OR 'disease management burden':ti,ab OR 'compliance burden':ti,ab))
23	TITLE-ABS-KEY(mortalit* OR death* OR "cause of death" OR "all-cause mortality" OR "disease-specific mortality" OR fatalit* OR surviv* OR "time to death")
24	TITLE-ABS-KEY(('health outcome' OR 'health outcome':ti,ab OR 'quality of life':ti,ab OR 'health status':ti,ab OR 'patient outcome':ti,ab OR 'treatment outcome':ti,ab OR 'clinical outcome':ti,ab OR 'disease outcome':ti,ab OR 'functional outcome':ti,ab OR 'health care outcome':ti,ab OR 'well-being':ti,ab) OR 'patient reported outcome measures':ti,ab OR 'patient reported outcome':ti,ab OR 'patient-reported outcome':ti,ab OR 'patient reported outcomes':ti,ab OR 'function':ti,ab OR 'pain' OR 'pain':ti,ab OR 'chronic pain' OR 'chronic pain':ti,ab OR 'health assessment questionnaire' OR 'health assessment questionnaire':ti,ab)
25	19 and 21 and 22 and 23 and 24
26	limit 25 to English language
